# Early and late signals of unexpected reward contribute to low extraversion and high disinhibition, respectively

**DOI:** 10.1017/pen.2021.4

**Published:** 2021-11-12

**Authors:** Phoebe S-H. Neo, Neil McNaughton, Martin Sellbom

**Affiliations:** Department of Psychology, University of Otago, Dunedin, New Zealand

**Keywords:** Reward-prediction-error, Personality, Dopamine, Anxiety, Impulsivity

## Abstract

Like socio-economic status and cognitive abilities, personality traits predict important life outcomes. Traits that reflect unusually low or high approach motivations, such as low extraversion and high disinhibition, are linked to various forms of mental disorder. Similarly, the dopamine system is theoretically linked to approach motivation traits and to various forms of mental disorder. Identifying neural contributions to extremes of such traits should map to neural sources of psychopathology, with dopamine a prime candidate. Notably, dopamine cells fire in response to unexpected reward, which suggests that the size of non-invasive, scalp-recorded potentials evoked by unexpected reward could reflect sensitivity in approach motivation traits. Here, we evaluated the validity of evoked electroencephalography (EEG) responses to unexpected reward in a monetary gain/loss task to assess approach motivation traits in 137 participants, oversampled for externalizing psychopathology symptoms. We demonstrated that over the 0–400 ms period in which feedback on the outcome was presented, responses evoked by unexpected reward contributed to all theoretically relevant approach motivation trait domains (disinhibition, extraversion and the behavioural activation system); and did so only at times when dopamine responses normally peak and reportedly code salience (70–100 ms) and valuation (200–300 ms). In particular, we linked “dopaminergic” salience and valuation to the psychopathology-related constructs of low extraversion (social anxiety) and high disinhibition (impulsivity) respectively, making the evoked potential components biomarker candidates for indexing aberrant processing of unexpected reward.

Poor mental health is a major global public crisis; and we urgently need to pinpoint the underlying brain dysfunctions. Despite this, our diagnostic manuals generally fail us with inadequate definitions of disorder (Krueger et al., 2018). Currently, we diagnose disorders via superficial symptoms and arbitrary thresholds. Each “disorder” is therefore, only weakly related to one or more underlying heterogeneous causes.

Recently, mental disorders have become linked to dimensional criteria (Cuthbert, 2014; Kotov, Krueger & Watson, 2018; Krueger et al., 2018; Sellbom, Ben-Porath & Bagby, 2008; Sellbom, Carragher, Sunderland, Calear & Batterham, 2020). That is, a mental disorder is not a discrete category but is an extreme of reactivities of our brain, mind and behaviour; and dysfunction is reflected in unusually high or low scores on dimensional criteria.

Like socio-economic status and cognitive abilities, personality traits predict important life outcomes (Roberts, Kuncel, Shiner, Caspi & Goldberg, 2007; Widiger et al., 2018). In particular, traits that reflect unusually low or high approach motivation, such as unusually low extraversion (aka introversion) or high disinhibition, are linked to various forms of mental disorders (Krueger & Tackett, 2003). Notably, personality traits are “transdiagnostic” dimensions. Identifying the neural basis of traits such as low extraversion and high disinhibition, would therefore allow us to pinpoint dysfunction-related brain reactivity, without the constraints of arbitrary definitions and thresholds (of mental disorders).

Extraversion includes personality facets of warmth, gregariousness, assertiveness, high energy, positive emotions and excitement seeking (Wilt & Revelle, 2017). Low extraversion (i.e. introversion), links to disturbances in mood, affect and anxiety, that is, “internalizing” problems, in particular, depression and social anxiety (Conway, Craske, Zinbarg & Mineka, 2016; Jylha & Isometsa, 2006; Naragon-Gainey & Simms, 2017; Tackett, Quilty, Sellbom, Rector & Bagby, 2008; Watson, Stasik, Ellickson-Larew & Stanton, 2015). Disinhibition includes facets of impulsivity, irresponsibility, distractibility, risk-taking, a lack of consideration for future consequences and past learning (Mullins-Sweatt, DeShong, Lengel, Helle & Krueger, 2019). High disinhibition links to “externalizing” problems (Choi et al., 2018; Krueger et al., 2009; Krueger & South, 2009; Mullins-Sweatt et al., 2019; Zisner & Beauchaine, 2015), for example, disruptive behaviours seen in antisocial personality disorder, conduct disorder, attention-deficit hyperactivity disorder (ADHD), and substance use disorder.

Dysfunctional dopamine reactivity has been theoretically linked to approach motivation traits (DeYoung, 2013; Wacker & Smillie, 2015; Zisner & Beauchaine, 2015), in particular, low extraversion and high disinhibition; and contributes to mental disorders (Ayano, 2016; Berry et al., 2019; Schneier et al., 2000; Zisner & Beauchaine, 2015), for example, depression, social anxiety and ADHD. An increase in dopamine activity could increase approach-motivation-related emotion, cognition and behaviours characterised by high extraversion and high disinhibition (Depue & Collins, 1999; DeYoung, 2013; Smillie et al., 2019; Zisner & Beauchaine, 2015). An increase in dopamine activity has also been theoretically linked to increased sensitivity in the neural system that mediates approach motivation (DeYoung, 2013) – that is, the behavioural activation system (Gray, 1982). The most common personality trait measure of the BAS (Carver & White, 1994) is associated in different ways with both extraversion and disinhibition (DeYoung, 2013).

In-depth analysis of dopamine cell firing in animals (Bromberg-Martin, Matsumoto & Hikosaka, 2010; Fiorillo, 2013; Matsumoto & Hikosaka, 2009) suggests that release of dopamine primarily signals unexpected reward – more specifically reward prediction error (RPE), the difference between a predicted and an experienced reward. The RPE is operationalized by observations that dopamine cell activation increases with unexpected reward, and decreases (from baseline) with omission of an expected reward (Morris, Arkadir, Nevet, Vaadia & Bergman, 2004; Schultz, Dayan & Montague, 1997). Importantly, current animal work (Redgrave & Gurney, 2006; Schultz, 2016; Schultz, Stauffer & Lak, 2017) reports two RPE signals: an early (70–100 ms) “salience” component; and a later (200–300 ms) “valuation” component. The early component detects, and orients us to, the occurrence of any unpredicted event (Redgrave & Gurney, 2006). The late component, as emphasized by Schultz (2016), codes the subjective motivational value of the unpredicted event (Schultz, 2016; Schultz et al., 2017).

Taken together, the above studies from parallel research areas suggest that extreme reactivities to RPE could reflect extreme brain sensitivities in some forms of psychopathology linked to approach motivation traits. Potts, Martin, Burton and Montague (2006) developed a gold bar/lemon task to test for human RPE signalling via event-related potentials recorded non-invasively from the human scalp. In the task, two sequential gold bars or two sequential lemons are usually presented and signal monetary reward, or non-reward, respectively. Occasionally, the second cue does not match the first and an unpredicted non-reward or reward occurs, respectively. Potts et al. (2006) found that in the 200–300 ms after the onset of the feedback stimulus, unexpected reward trials showed a less negative potential than that of unexpected non-reward trials, as have others (Cooper, Duke, Pickering & Smillie, 2014; Smillie, Cooper & Pickering, 2011; Smillie et al., 2019; Walsh & Anderson, 2012). When the event-related potentials in the unexpected reward trials were contrasted with the unexpected non-reward trials (i.e. the non-reward case subtracted from the reward), to estimate RPE, a larger positive RPE response was associated with higher levels of extraversion (Cooper et al., 2014; Smillie et al., 2011, 2019).

Existing work suggests that the human scalp RPE shows characteristics consistent with animal depth recorded dopamine response (Ullsperger, Fischer, Nigbur & Endrass, 2014); and support theoretical links (DeYoung, 2013; Wacker & Smillie, 2015; Zisner & Beauchaine, 2015) between brain dopamine and approach motivation traits. However, an attempt to show a positive relation of RPE to the BAS failed to detect any reliable effects (Cooper et al., 2014). While the previous human work found a positive relationship of RPE to extraversion, it has not explicitly linked RPE to disinhibition. Furthermore, it analysed RPE only in the 200–300 ms feedback period.

Therefore, in this study, using the gold bar/lemon task, we tested the hypothesis that both early (70–100 ms) and late (200–300 ms) phasic responses to RPE would show positive correlations with trait measures of disinhibition, as well as extraversion and the BAS. As the dopamine system primarily codes unexpected reward and not expected reward (Eshel, Tian, Bukwich & Uchida, 2016), we also hypothesized that signals of expected reward would not show the same pattern of correlations as that generated by RPE/unexpected reward. In addition, we used a sample over-weighted towards externalizing psychopathology. This sample had variability in both RPE and trait features that might have been restricted in a non-externalizing sample; with the restriction suppressing correlations.

## 1. Methods

### 1.1. Participants

Participants were recruited as part of a larger study via Facebook advertisements and flyers in the community designed to target a sample that was over-weighted towards externalizing psychopathology. Ethical approval was provided by the University of Otago Ethic Committee (Health), Approval number: H16/031. Gold bar/lemon task data were available for 160 participants (71 males, 89 females; aged 18–56 years; mean = 37; SD = 9). Twenty-three of these participants were excluded due to invalid MMPI-3 scores (based on excessive inconsistent or otherwise deviant responding per the MMPI-3 manual (Ben-Porath & Tellegen, 2020) and/or loss of EEG data due to excessive noise/artefact. The final sample consisted of 137 participants including 57 males and 80 females. Age range and mean were unchanged.

As expected from the overweighting towards externalizing problems, per structured clinical interviews, 51.8% of the sample met diagnostic criteria for at least one DSM-5 externalizing disorder, with ADHD (any type, 26%), history of conduct disorder (22%), alcohol use disorder (21%), antisocial personality disorder (17.5%) and cannabis use disorder (12%) being the most frequent. These rates are far higher than a typical community sample (Kessler et al., 2011), reflecting the externalizing nature of participants (see Table S1 in the online supplementary material for a summary of the means and ranges of the personality scores in the current sample).

### 1.2. The gold bar/lemon task

**Figure 1.**
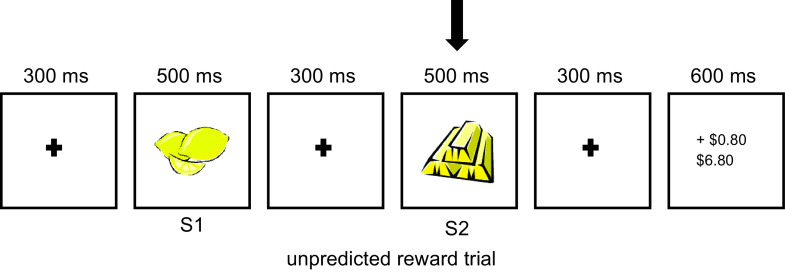
Sequence of events in an unpredicted reward trial. S1 indicates the first stimulus onset and S2 indicates the second stimulus onset. S1 and S2 were always either a lemon or a bar. The period of interest for the current study is indicated by the vertical arrow.

The task was delivered via ePrime (version 2.0; Psychology Software Tools, Inc. Sharpsburg, PA, USA) using code provided by Dr L. Smillie. It consisted of four types of trials, differentiated by the presentation sequence of two stimuli, S1 or S2, which could be either a lemon or a gold bar. In a predicted reward (PR) trial, S1 and S2 were both gold bars. In a predicted non-reward (PNR) trial, S1 and S2 were both lemons. Figure 1 shows the sequence and the timing of events in an unpredicted reward (UR) trial, where S1 was a lemon and S2 was a gold bar. In an unpredicted non-reward (UNR) trial, S1 and S2 were switched compared with UR. All other parameters were the same for all types of trial. There were 30 practice trials; followed by eight blocks of 60 real trials. S1 consisted of a lemon or a gold bar half the time. 20% of the trials consisted of a mismatching S2. In the practice trials, participants made a keypress to start each trial. During the actual trials, no keypress was required and participants were instructed to simply pay attention to the game. Participants were provided with $6 at the start of the game; and NZD 0.20 was deducted from each trial. In the reward trials, participants were paid NZD $1. Inter-trial intervals were irregular, ranging from 2000 to 3600 ms, with a “blink now” message displayed. Participants were instructed to avoid blinking at other times to reduce artefacts. Participants were told that they would receive the total earnings from the block of trials that produced the highest amount of earnings. The monetary outcomes were fixed and hence the same across participants. This was fixed at $6 for all the participants.

### 1.3. EEG acquisition and processing

EEG was recorded referenced to CPz and sampled at 512 Hz from FP1, FPz, FP2, F7, F3, Fz, F4, F8, T7, T8 C3, Cz, C4, P7, P3, Pz, P4, P8, M1 and M2 with an advanced neuro technology (ANT) amplifier and ANT caps with AgCl electrodes. Impedances were tested with ANT software (eego) and kept below 10 kΩ. Matlab 2019a plugins, EEGlab (version 2019_0) and ERPlab (version 7.0.0) were used to process the data. The EEG was first filtered (pop_eegfiltnew) with a cut off at 100 Hz then re-referenced to the average of all the recording electrodes excluding the mastoid electrodes. EEG epochs were extracted from S2 − 1100 ms through to S2 + 1400 ms (arrow in Figure 1 indicates S2). Linear drift was removed from each such epoch using pop_eeglindetrend and then reduced to S2 − 100 ms through S2 + 400 ms and baseline corrected using the average of S2 − 100 ms to S2. Epochs with a range greater than 70 µV were then rejected (pop_artextval). Participants with fewer than 20 UR or UNR trials left for averaging after the artefact rejection step were excluded from further analyses.

### 1.4. EEG measures

RPEs were estimated by subtracting event-related potentials in the UNR trials from the UR trials. The periods of primary interest for analysis were: 70–100 and 200–300 ms (Redgrave & Gurney, 2006; Schultz, 2016; Schultz et al., 2017), where activity of the key phasic dopamine components peaked. To examine time course, we also extracted the intermediate time periods with the full set of analysed time periods being: 0–70 ms (t1), 70–100 ms (t2), 100–200 ms (t3), 200–300 ms (t4) and 300–400 ms (t5). Signals of expected reward were estimated by subtracting event-related potentials in the PNR trials from the PR trials.

All analysis was restricted to the electrode site Fz, where RPE was previously identified in the gold bar/lemon task (Cooper et al., 2014; Smillie et al., 2011, 2019; Walsh & Anderson, 2012).

### 1.5. Personality and psychopathology measures

We measured approach motivation traits with three self-report inventories: the Eysenck Personality Questionnaire – Revised (EPQ-R) (Eysenck & Eysenck, 1994); Carver and White’s (1994) Behavioural Inhibition/Behavioural Activation System (BIS/BAS) scales; and the Minnesota Multiphasic Personality Inventory.

The EPQ-R (Eysenck & Eysenck, 1994) is a 103-item self-report inventory. Participants make a true/false binary choice on each item. The items aggregate onto 4 scales, developed to measure biological traits at the broader, domain level. We used only extraversion for personality assessment, to facilitate comparison across studies – a positive relation between EPQ-R extraversion and RPE was reported previously (Cooper et al., 2014; Smillie et al., 2011, 2019).

Carver and White (1994) developed the BIS/BAS scale to measure two neural motivation systems (Gray, 1970), namely the Behavioural Inhibition System (BIS), and the Behavioural Activation System (BAS). The BIS/BAS scale is a 24-item self-report inventory. Participants respond on a 4-point Likert scale that reflects the degree an item is true or false. The items aggregate onto 4 scales. We used a BAS total score as well as the three subscales (drive, reward sensitivity and fun seeking) designed to measure BAS sensitivity. These showed good alignment with the theoretical construct of BAS sensitivity (Krupić, Corr, Ručević, Križanić & Gračanin, 2016).

The Minnesota Multiphasic Personality Inventory −3 (MMPI-3) (Ben-Porath & Tellegen, 2020) is a well-validated self-report inventory designed to assess a range of psychopathology constructs and maladaptive personality traits in accordance with contemporary theories of psychopathology and personality. It has representative population norms derived from the general community and allow us to assess psychopathological manifestations of extraversion and disinhibition in the general population. It includes 335 items to which participants responded true or false as items applied to them. The MMPI-3 items aggregate onto 10 validity scales and 42 scales that measure substantive content. Among these latter scales, we specifically used low positive emotions, introversion/low positive emotionality, shyness, social avoidance, disaffiliativeness and dominance for assessment of extraversion/introversion traits. Several scales also measure disinhibited-externalizing including antisocial behaviour, juvenile conduct problems, substance abuse, impulsivity and disconstraint (Sellbom, 2020). Hypomanic activation and its facet scale, activation, were also included as they assess a combination of disinhibition and excessive psychological energy consistent with dopaminergic activity (DeYoung, 2013).

### 1.6. Procedure

All participants took part in the experiment as part of a larger study. Upon arrival at the laboratory, they completed a battery of questionnaires that included the EPQ-R, MMPI-3 and BIS/BAS scales while the experimenter applied electro-gel and reduced the sensors’ impedance on their EEG cap. The participants were then tested (˜25 min; data not analysed here) on the stop-signal task (Shadli et al., 2020), followed by the gold bar/lemon task (˜30 min). After the EEG tests, the participants were administered structured clinical interviews for a different study (data not analysed here). The experimental session was conducted by a trained research assistant under supervision by a registered clinical psychologist. At the end of the experiment, participants were paid $50 in petrol or supermarket vouchers, and cash winnings ($6) from the gold bar/lemon task.

## 2. Results

**Figure 2.**
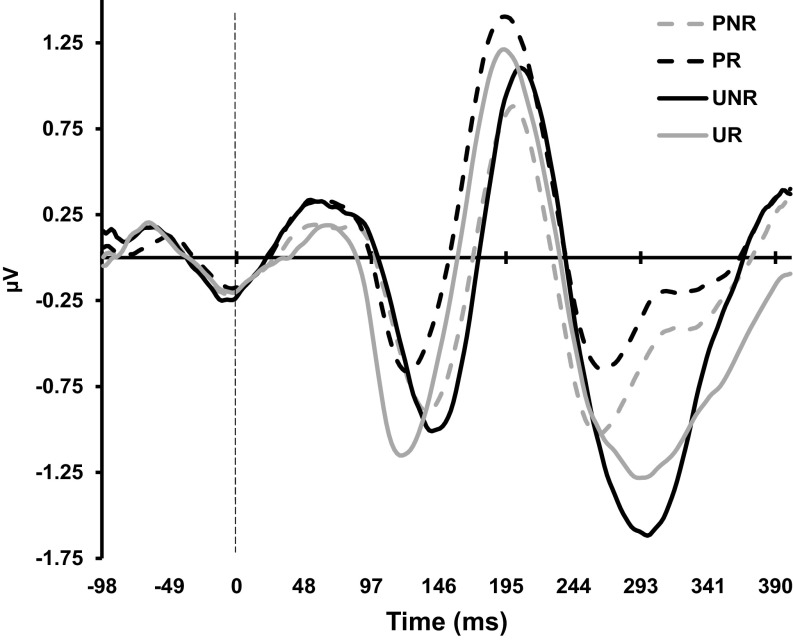
Event-related potentials for each of the four experimental conditions in the gold bar/lemon task. PNR: Predicted Non-Reward; PR: Predicted Reward; UNR: Unpredicted Non-Reward; UR: Unpredicted Reward.

Event-related potentials averaged across trials, for each of the four experimental conditions, are shown in Figure 2. As in previous studies (Cooper et al., 2014; Smillie et al., 2011, 2019), we observed negative peaks in the 200–300 ms period, in all of the conditions. But ours were somewhat later: UNR peaked at 300 ms versus ˜280 ms previously (Cooper et al., 2014; Smillie et al., 2011, 2019), with the other negative peaks similarly shifted in time (i.e. ˜+20 ms).

**Figure 3.**
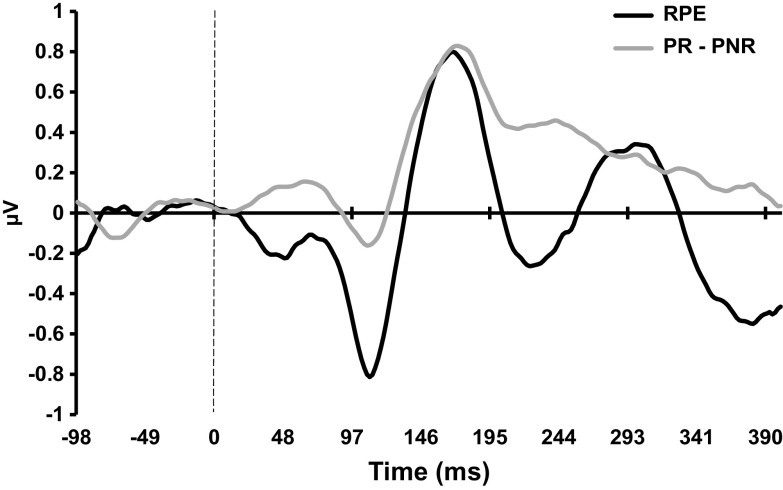
Event-related potentials for reward-prediction-errors (RPE, black) and expected reward (PR - PNR, grey). RPE was calculated by subtracting event-related potentials of unexpected non-reward trials from unexpected reward trials. PNR: Predicted Non-Reward; PR: Predicted Reward; UNR: Unpredicted Non-Reward; UR: Unpredicted Reward.

As shown in Figure 3, the RPE contrast (UR – UNR) had two positive peaks as in previous studies (Cooper et al., 2014; Smillie et al., 2011). Previously, the second positive peak was larger than the first. However, here, the trend was reversed – largely due to a smaller second peak. Figure 3 also shows the contrast for expected reward (PR – PNR), which showed a positive peak of similar amplitude and time course to that of the first positive RPE peak.

Pearson correlations between the trait measurements and RPE components from t1 to t5 are shown in Table 1. Statistically significant correlations were observed only at t2 and t4. Unique correlations for t2 were MMPI-3 shyness (negative) and EPQ-R extraversion (positive), and for t4, was BAS-reward responsivity (positive). The remaining significant correlations for t4 were shared with t2 (all positive); and included MMPI-3 impulsivity, BAS-drive and BAS-total.

**Table 1.** Pearson correlations for reward-prediction-errors (RPE)/unexpected reward

RPE	t1	t2	t3	t4	t5
Low Positive Emotions	−.02	−.05	−.05	−.04	.03
Introversion/Low Positive Emotionality	−.13	−.02	−.07	−.05	−.03
Shyness	−.06	−.16*	−.06	−.05	−.07
Social Avoidance	−.12	−.03	−.06	.00	.05
Disaffiliativeness	−.05	−.03	.07	−.03	−.03
Dominance	−.06	.11	.05	−.02	−.06
Extraversion (EPQ-R)	.10	.21**	.03	.03	−.07
Hypomanic Activation	.04	.14	.09	.11	.01
Activation	−.01	.08	.08	.10	−.04
Juvenile Conduct Problems	.06	.10	.10	.11	.05
Substance Abuse	.01	−.03	.01	−.05	−.01
Impulsivity	.12	.18*	.08	.16*	.05
Disconstraint	.07	.03	.03	.04	.02
Antisocial Behaviour	.03	−.04	.05	.01	−.01
BAS Drive (C&W)	.07	.18*	.13	.15*	.01
BAS Fun Seeking (C&W)	.10	.12	.08	.10	−.02
BAS Reward Responsivity (C&W)	−.01	.08	.10	.16*	.02
BAS Total (C&W)	.07	.17*	.14	.18*	.00

* *p < 0.05*; ** *p < 0.005*, one-tailed Pearson correlations, uncorrected. Unless otherwise stated in brackets, traits are MMPI-3 measures. Note that low/negative scores on the following traits denote high extraversion (broadly defined): Shyness; Social Avoidance and Disaffiliativeness. t1: 0–70ms; t2: 70–100ms; t3: 100–200ms; t4: 200–300ms; t5: 300–400ms.

Pearson correlations between the trait measurements and the expected reward components from t1 to t5 are shown in Table 2. In contrast to RPE, statistically significant correlations were not detected at t2 and t4. Instead, we observed reliable negative correlations at t1 for MMPI-3 disaffiliativeness; MMPI-3 hypomanic activation; BAS-fun seeking and BAS-total. Notably, BAS-fun seeking also showed reliable negative correlates at t3 and t5.

**Table 2.** Pearson correlations for expected reward

RPE	t1	t2	t3	t4	t5
Low Positive Emotions	.01	−.03	.02	.02	.03
Introversion/Low Positive Emotionality	−.06	.02	.03	−.01	.00
Shyness	−.03	−.03	−.05	.02	.07
Social Avoidance	−.11	−.01	−.03	−.07	−.03
Disaffiliativeness	−.20**	−.03	.07	.01	−.02
Dominance	.01	.02	.05	.02	−.01
Extraversion (EPQ-R)	.03	.02	.05	.06	.06
Hypomanic Activation	−.14*	−.13	−.08	−.02	−.07
Activation	−.09	−.10	−.09	−.03	−.07
Juvenile Conduct Problems	−.03	.00	.10	.01	−.05
Substance Abuse	.01	−.04	.05	.00	−.08
Impulsivity	−.11	−.12	−.11	−.04	−.12
Disconstraint	−.05	−.06	.01	−.01	−.08
Antisocial Behaviour	−.04	−.01	.08	.01	−.08
BAS Drive (C&W)	−.10	.08	−.05	−.06	−.02
BAS Fun Seeking (C&W)	−.20*	−.10	−.16*	−.10	−.16*
BAS Reward Responsivity (C&W)	−.06	−.04	−.05	.01	.07
BAS Total (C&W)	−.16*	−.02	−.11	−.07	−.05

* *p < 0.05*; ** *p < 0.005*, one-tailed Pearson correlations, uncorrected. Unless otherwise stated in brackets, traits are MMPI-3 measures. Note that low/negative scores on the following traits denote high extraversion (broadly defined): Shyness; Social Avoidance and Disaffiliativeness. t1: 0–70ms; t2: 70–100ms; t3: 100–200ms; t4: 200–300ms; t5: 300–400ms.

## 3. Discussion

In this study, we demonstrated that all theoretically relevant trait domains of approach motivation (disinhibition, extraversion and the BAS) were positively related to RPE, that is, unexpected reward. Importantly, the trait–RPE associations were observed at time points when dopamine activity normally peaks in animal studies (Redgrave & Gurney, 2006; Schultz, 2016). Specifically, correlation of the traits with RPE were observed in t2 (70–100 ms) and t4 (200–300 ms), but not in the intermediate periods. Also consistent with the assignment by Schultz (2016) of distinct functional roles to the early (salience) and late (valuation) peaks, we observed some distinct trait–RPE associations for the two times. At 70–100 ms, RPE uniquely correlated with MMPI-3 shyness (negative) and EPQ-R extraversion. At 200–300 ms, RPE uniquely correlated with BAS-reward responsivity. MMPI-3 impulsivity (a variant measure of disinhibition), BAS-drive and BAS-total, correlated with RPE components at both times. In contrast, the association of expected reward signals with the traits did not follow a “dopaminergic” time course, reinforcing the possibility that dopamine mediated the associations of the traits with RPE.

Available human dopamine neuron data (Zaghloul et al., 2009) are also broadly consistent with the animal results, showing a clear RPE response in the region of 200–250 ms. To our knowledge, human scalp-recorded RPE in the 70–100 ms range has not been examined previously. We extended previous work (Smillie et al., 2011, 2019; Walsh & Anderson, 2012) by showing that human scalp-recorded RPE potentials are like depth activity in animals (Redgrave & Gurney, 2006; Schultz, 2016), with an early component that, like the late, 200–300 ms, RPE component, contributes to approach motivation traits.

We also found support for the existing positive correlation between extraversion and RPE (Cooper et al., 2014; Smillie et al., 2011, 2019), albeit at the early not the late time (at t2 rather than at t4). We have also noted other differences in the time course and size of the RPEs (see the Results section). Recruitment of a sample over-weighted for externalizing problems, in contrast to a normal sample, could account for the difference. In addition, the gold bar/lemon task was administered after the stop-signal task. Boredom and fatigue could have set in and altered the RPE responses somewhat; as could a different relative value of the dollar payment for this group.

It should be noted that previously, Cooper et al. (2014) hypothesized but failed to detect, a positive relationship between RPE and each of the three Carver and White BAS scales in the gold bar/lemon task. We reported reliable positive relations of RPE with BAS-total; BAS-drive and BAS-reward responsivity, respectively, and this difference could be due to a lack of statistical power from their smaller sample size (*n* = 38), or a restricted range of scores (in light of their sampling). Indeed, their observed effect sizes were not dissimilar from ours. The *r* values in Cooper et al. (2014) were: BAS-drive, *r* = −.1; BAS-reward responsivity, *r* = .22; BAS-total was not reported.

Aberrant processing related to approach motivations, which has featured consistently in psychopathology, still tends to be studied within the framework of categorical disorders (Alloy, Olino, Freed & Nusslock, 2016; Gold, Waltz, Prentice, Morris & Heerey, 2008; Proudfit, 2015; Volkow et al., 2010). Here, we demonstrate the utility of studying the biological basis of dimensional trait measures for understanding psychopathology-related traits. Our evidence implicates distinct “dopaminergic” RPE components in different facets of approach motivation traits, consistent with recent work suggesting that aberrant approach-motivation-related processing can manifest at distinct temporal stages and subcomponents (Bowyer et al., 2021). In particular, MMPI-3 shyness measures social anxiety, which is linked clinically to low extraversion (Sellbom et al., 2020). Our results therefore implicate the “dopaminergic” orienting response as a potential source of impairment in social anxiety. Similarly, special consideration of RPE relations with impulsivity in both the early and late phases suggests two potential sources of “dopaminergic” dysfunctions in highly disinhibited individuals: 1) they may be hypervigilant to the presence of possible unexpected reward; and 2) may also overvalue unexpected reward.

Our findings above are consistent with existing work that reports dopamine system irregularities in mental disorders, such as social anxiety and ADHD (Ayano, 2016; Berry et al., 2019; Schneier et al., 2000; Zisner & Beauchaine, 2015). We extended the existing knowledge by pinpointing the psychological roles (salience versus valuation) and temporal dynamics that could be involved.

Regrettably, the small number of EEG sensors used here did not allow for reliable source localization; and the suggested involvement of the dopamine system, although highly plausible considering the temporal dynamics observed here, remains tentative. The effect sizes were also small and correlational in nature. We cannot rule out the possibility of type I errors either as we conducted a large number of analyses, but ultimately did not have sufficient power for substantial corrections due to family-wise error owing to the small effect sizes typically observed in these research studies. Hence, it is important that future studies replicate the findings. Nonetheless, it is also worth noting that the findings are not inconsistent with previous work on personality theories and RPE processing (Beauchaine, Zisner & Sauder, 2017; DeYoung, 2013; Schultz et al., 2017, 2019; Wacker & Smillie, 2015).

Sixty-four EEG sensors are recommended for source localization analyses (Sohrabpour et al., 2015). A replication of the results with such a system would also allow for functional connectivity analyses, and provide us with an in-depth picture of the neural circuitry that could be involved. However, we suggest that future studies use a version of the task that requires active engagement by the participants to increase signal intensity.

Future work could also test for a causal relationship between impaired “dopaminergic” salience processing and anhedonia. Social anxiety includes components of anhedonia and anxiousness (Brown, Chorpita & Barlow, 1998; Kashdan, 2007; Kashdan, Weeks & Savostyanova, 2011). If the dopamine system was indeed involved, we predict that anhedonia in socially shy participants was the underlying cause of the relationship between salience processing and shyness observed here. It follows that we would then observe hypo-reactivity to “dopaminergic” salience in patients that exhibit anhedonia across disorders where this normally manifests, that is, social anxiety as well as depression.

Similarly, impulsivity is a feature in many externalizing disorders, such as substance abuse and ADHD (Krueger & South, 2009; Patrick et al., 2013). Further work to ascertain if it is indeed only over-reactivity to unexpected, and not expected reward processing that contributes to impulsivity, would help to further pinpoint its psychological and neural sources.

Last, but not least, although the effect sizes in this study were small, the pinpointing of potential psychological and neural sources is a significant step for achieving a more precise understanding of aberrant reward processing. Furthermore, we can test for scalp-recorded RPE signals at a low cost and non-invasively, making them prime candidates for “dopaminergic” biomarkers of hypo-/hyper-reactivity to aberrant processing of unexpected reward. Assessing their validity as biomarkers in psychopathology is therefore a key direction for future work.

## Supplementary material

To view supplementary material for this article, please visit https://doi.org/10.1017/pen.2021.4. click here to view supplementary material
