# Supplementary material for: Early and late signals of unexpected reward contribute to low extraversion and high disinhibition, respectively
Source: Personal Neurosci. 2021 Nov 12;4:e5. doi: 10.1017/pen.2021.4 (PMC8645529; doi:10.1017/pen.2021.4)
Supplement: Supplementary file 1 [file S2513988621000043sup001.pdf]

Supplementary materials

Table S1. Minimum, maximum, means and standard deviations for the trait measures.

Trait measures	Min	Max	Mean	SD
Low Positive Emotions	36.20	82.20	55.60	10.60
Introversion/Low Positive Emotionality	34.90	80.90	54.01	12.10
Shyness	37.70	76.60	53.69	10.00
Social Avoidance	36.90	77.20	52.91	11.70
Disaffiliativeness	39.60	77.90	51.87	11.30
Dominance	27.00	69.30	47.08	9.69
Extraversion (EPQ-R)	3.00	23.00	13.17	5.38
Hypomanic Activation	32.10	85.80	53.70	12.30
Activation	35.20	79.70	52.22	10.40
Juvenile Conduct Problems	38.80	80.50	55.69	12.90
Substance Abuse	39.20	85.60	59.21	14.30
Impulsivity	37.10	75.50	55.23	13.70
Disconstraint	34.20	85.50	58.73	14.50
Antisocial Behavior	34.60	86.10	58.06	13.30
BAS Drive (C&W)	1.00	4.00	2.81	0.64
BAS Fun Seeking (C&W)	1.76	4.00	3.06	0.56
BAS Reward Responsivity (C&W)	2.20	4.00	3.40	0.46
BAS Total (C&W)	2.00	3.86	3.11	0.40

Unless otherwise stated in brackets, traits are Minnesota Multiphasic Personality Inventory -3 (MMPI-3) measures. EPQ-R: The Eysenck Personality Questionnaire – Revised; BAS: Carver and White Behavioural Activation System Scale. All MMPI-3 scores are expressed in T-score units.
